# Successful conservative treatment of contralateral recurrent ectopic pregnancy with high β-HCG after unilateral salpingectomy: a case report

**DOI:** 10.3389/fmed.2026.1916585

**Published:** 2026-08-03

**Authors:** Jiali Ruan, Yue Hu, Xuelian Du

**Affiliations:** 1 Graduate School, Guangzhou University of Chinese Medicine, Guangzhou, Guangdong, China; 2 Graduate School, The Fourth Clinical Medical College of Guangzhou University of Chinese Medicine, Shenzhen, China; 3 Department of Gynecology, Shenzhen Hospital of Traditional Chinese Medicine, Shenzhen, China

**Keywords:** case report, conservative treatment, ectopic pregnancy, maternity protection, methotrexate

## Abstract

**Background:**

Ectopic pregnancy (EP) is a common gynecological emergency, and its clinical management requires comprehensive consideration of β-human chorionic gonadotropin (β-HCG) level, mass size, fertility needs, and vital signs. Methotrexate (MTX)-based conservative treatment is mostly suitable for stable patients with β-HCG < 5,000 IU/L and no active bleeding, while surgical treatment is routinely recommended for those with β-HCG > 5,000 IU/L.

**Case presentation:**

This article reports a 30-year-old patient with ectopic pregnancy (G4P0) who had a history of laparoscopic left salpingectomy due to ectopic pregnancy and two previous artificial abortions. The initial β-HCG level on admission was 5,823 IU/L, which later increased to 6,609 IU/L. The patient had stable vital signs but a strong demand for fertility preservation. She refused surgical treatment and received combined traditional Chinese and Western medicine conservative treatment. After the first MTX administration, β-HCG increased to 8,516 IU/L, and then gradually decreased after continued conservative treatment. At discharge, β-HCG decreased to 1,067.0 IU/L, and follow-up in the outpatient clinic showed that β-HCG turned negative. Conservative treatment was successful without severe complications such as ectopic pregnancy rupture or massive hemorrhage.

**Conclusions:**

This case demonstrates that in a carefully selected patient with stable vital signs, absence of rupture, strong fertility preservation desire, and good compliance, conservative management with repeated MTX doses may be a feasible alternative to surgery even when initial β-hCG exceeds 5,000 IU/L. However, this approach requires strict patient selection, intensive monitoring, and preparedness for emergency surgical conversion. The individual contribution of Traditional Chinese Medicine (TCM) to the successful outcome cannot be ascertained from this single case.

## 1 Introduction

Ectopic pregnancy (EP) remains a leading cause of early pregnancy-related mortality worldwide (1). Methotrexate (MTX) is the cornerstone of medical management for early unruptured EP; however, its success rate is inversely correlated with baseline β-hCG levels (2, 3). When β-hCG exceeds 5,000 IU/L, MTX failure rates are reported to reach 14.3%−62% (4, 5), prompting major international guidelines, including those from CNGOF and ACOG, to recommend surgical intervention as the preferred primary treatment in such scenarios (5, 6). In contrast, NICE and RCOG have adopted more conservative thresholds, recommending that methotrexate be used primarily when β-hCG levels are < 1,500 IU/L (7, 8).

The clinical challenge intensifies significantly in patients presenting with recurrent ectopic pregnancy (REP) after prior contralateral salpingectomy. REP occurs in approximately 10%−27% of cases following an initial EP (9), and in the context of a single remaining fallopian tube, fertility preservation becomes a paramount concern. Unfortunately, there is a notable paucity of evidence-based guidance specifically addressing medical management in this high-risk subgroup—namely, those with β-hCG > 5,000 IU/L and a solitary tube. Recently, novel prognostic tools, such as the scoring system proposed by Kale et al. (10) for predicting single-dose MTX success , have advanced our ability to select appropriate candidates for medical therapy; nevertheless, real-world data from complex, high-risk presentations remain scarce.

Herein, we report a rare case of a patient with a history of unilateral salpingectomy who presented with a recurrent tubal ectopic pregnancy and an initial β-hCG exceeding 5,000 IU/L. She was successfully managed with a modified repeated-dose MTX regimen combined with adjunctive Traditional Chinese Medicine (TCM) therapies and intensive surveillance. Given the uncontrolled, single-case nature of this observation, we explicitly do not attribute the successful outcome to any single intervention. Rather, we aim to explore the feasibility, key decision-making checkpoints, and strict monitoring prerequisites for individualized conservative management in such exceptional circumstances, with the goal of providing a practical reference for clinicians encountering similar challenging cases, while fully acknowledging the limitations of our approach.

## 2 Case presentation

### 2.1 Medical history and examination

A 30-year-old nulliparous woman (gravida 4, para 0) presented to our department with an 8-day history of right-sided lower abdominal pain and mild vaginal spotting. Her last menstrual period was 8 weeks prior. She had a history of left salpingectomy for tubal ectopic pregnancy 3 years ago. There was no history of pelvic inflammatory disease, intrauterine device use, or assisted reproductive technology. She was a non-smoker with no significant past medical history.

On admission, vital signs were stable: blood pressure 109/70 mmHg, heart rate 81 beats/min, respiratory rate 20/min, temperature 36.2 °C. Abdominal examination revealed that the entire abdomen is soft without guarding or rebound tenderness. Gynecological examination: Vagina patent; slight bleeding originating from the uterine cavity observed; cervix smooth, with no pain on flexion or extension; uterus retroverted, normal size, of moderate consistency, mobile, and without tenderness; right adnexa thickened, without tenderness; no abnormalities noted in the left adnexa.

**Figure 1:**
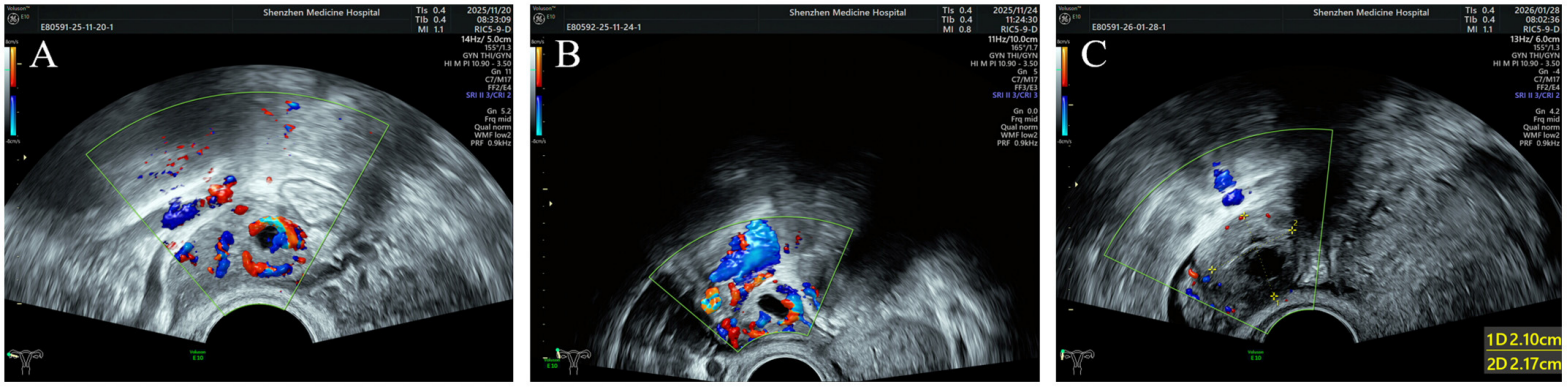
Transvaginal ultrasound images before, during, and after treatment. **(A)** 2025-11-20 First ultrasound after admission: a mixed echo was seen next to the right ovary, about 13 × 13 mm in size. **(B)** 2025-11-24 Ultrasound after the first MTX treatment: a mixed echo was seen next to the right ovary, about 16 × 13 mm in size. **(C)** 2026-01-28 Ultrasound when HCG turned negative: a mixed echo was seen next to the right ovary, about 22 × 21 × 22 mm in size.

*Ultrasound findings*: (1) No obvious gestational sac was observed in the uterine cavity (endometrial thickness: 10.4 mm); (2) Mixed-echo lesion adjacent to the right ovary (approximately 13 × 10 mm in size, with a small hypoechoic area visible within; CDFI: abundant blood flow signals visible around the periphery); possible ectopic pregnancy mass to be ruled out; (3) Possible corpus luteum in the right ovary (18 × 12 mm mixed-echo lesion with a ring-shaped blood flow signal around the periphery), O-RADS 2; (4) No obvious dark areas indicative of free fluid were observed in the pelvis (Figure 1).

*Laboratory findings*: Serum β-hCG on admission was 5,823 IU/L. Complete blood count showed no abnormalities (hemoglobin 119 g/L). Liver function tests were within normal limits at baseline (ALT 10.6 U/L, AST 17 U/L). Renal function and coagulation profile were normal.

## 2.2 Treatment and management

After thorough counseling regarding the risks of conservative treatment—including treatment failure, tubal rupture, need for emergency surgery, and potential loss of the remaining fallopian tube—the patient expressed a strong desire to preserve her fertility and opted for medical management. Written informed consent was obtained. The chronological timeline of Management is shown in Table 1.

**Table 1:** Chronological timeline of management.

Day	β-hCG (IU/L)	Intervention	Ultrasound findings	Adverse events
The day of admission	5,823	Surgery was recommended, but the patient refused and requested conservative treatment	No gestational sac in the uterine cavity; mixed echoes beside the right ovary (13 × 10 mm) with abundant peripheral blood flow signals (considering ectopic pregnancy mass)	None
Day 1 of conservative treatment	6,609	MTX 50 mg/m^2^ IM; TCM initiated	A 13 × 13 mm hyperechoic mass near the right ovary; 20 mm of pelvic effusion	None
4	8,516	MTX 50 mg/m^2^ IM (2nd dose)	A 16 × 13 mm hyperechoic mass near the right ovary; 20 mm of pelvic effusion	None
7	7,200	MTX 50 mg/m^2^ IM (3rd dose)	–	ALT 44.1 U/L (normal range: 7–40), AST 40.5 U/L (normal range: 13–35)
12	3,440	Hepatoprotective agents started	Mass stable 19 × 15 mm; minimal free fluid (< 0.5 cm)	ALT 160.1 U/L, AST 132.3 U/L (peak)
18	1,067	Discharge	Mass 27 × 24 × 19 mm; 14 mm of pelvic effusion	ALT 36.3 U/L, AST 23.3 U/L; Liver enzymes normalized
26	701	Continued hepatoprotection	Mass 26 × 24 × 19 cm	ALT 61.1 U/L, AST 50.2 U/L
36	266	Continued hepatoprotection	Mass 27 × 35 × 19 mm	ALT 107.8 U/L, AST 75.6 U/L
2 months later	< 0.1 (negative)	–	Mass 22 × 21 × 22 mm	ALT 24.0 U/L, AST 25.8 U/L; Liver enzymes normalized

MTX, methotrexate; IM, intramuscular; TCM, traditional Chinese medicine; ALT, alanine aminotransferase; AST, aspartate aminotransferase.

*Rationale for three MTX doses*: The patient was initially started on a modified 2-dose MTX protocol (50 mg/m^2^ on day 1 and day 4). However, because β-hCG levels actually rose from 6,609 IU/L to 8,516 IU/L on Day 4, and the decrease in β-hCG between Days 4 and 7 was less than 15%, an additional dose was administered on Day 7. This approach is consistent with the flexible extension of the 2-dose protocol described in the literature (11), in which up to four doses may be safely administered within 2 weeks in selected patients.

*Integrated TCM interventions*: The patient concurrently received modified Gongwaiyun No. II Decoction (a TCM formula designed to promote blood circulation, remove blood stasis, eliminate masses, and exert embryocidal effects), oral Chinese patent medicines (Sanjie Zhentong Capsules), and external TCM applications. Infrared irradiation was also applied to the lower abdomen for symptomatic relief. We explicitly acknowledge that, given the simultaneous administration of multiple interventions, the individual contribution of TCM to the successful outcome cannot be determined from this single case.

*Adverse events and management*: Transient elevation of liver enzymes was noted from day 7, with peak values of ALT 160.1 U/L and AST 132.3 U/L. Hepatology consultation was obtained, and hepatoprotective agents (including bicyclol and reduced glutathione) were administered. Liver enzymes gradually declined and returned to normal. No other significant adverse events occurred. The patient remained hemodynamically stable throughout the treatment course, with no signs of tubal rupture.

### 2.3 Treatment outcome and follow-up

During hospitalization, the patient received 3 doses of MTX treatment combined with an integrated traditional Chinese and Western medicine regimen. No severe complications such as ectopic pregnancy rupture, massive hemorrhage, or severe adverse drug reactions occurred, and vital signs remained stable. At discharge, β-HCG was 1,067.0 IU/L, and liver and kidney function was normal (ALT 36.3 U/L, AST 23.3 U/L).

**Figure 2:**
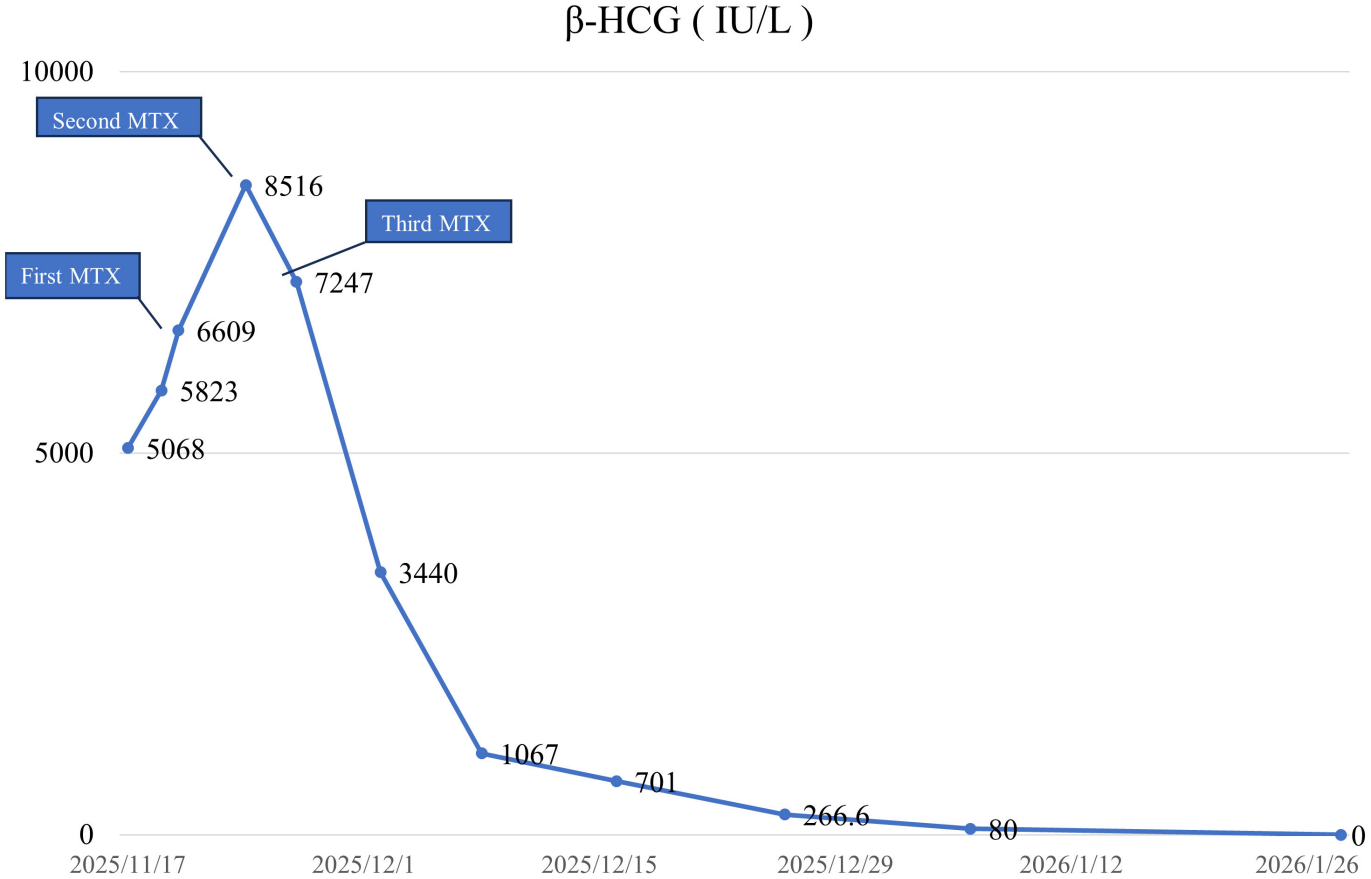
β-HCG change curve.

Continuous follow-up after discharge showed that β-HCG gradually decreased to negative (Figure 2); mild elevation of liver enzymes occurred during follow-up, which returned to normal after liver protection treatment; ultrasound suggested that the mixed echo mass beside the right ovary was stable (Figure 1), with no signs of intra-abdominal bleeding, and conservative treatment was successful.

## 3 Discussion

This case describes the successful conservative management of a recurrent tubal ectopic pregnancy with initial β-hCG > 5,000 IU/L in a patient with prior contralateral salpingectomy. While the clinical outcome was favorable, several important considerations warrant discussion regarding the interpretation of this case and its implications for clinical practice.

### 3.1 β-hCG threshold and predictors of MTX success

The efficacy of methotrexate (MTX) is inversely correlated with baseline β-hCG levels (3, 12). However, no universally accepted threshold exists. Different guidelines and studies use different thresholds based on the characteristics of their populations and clinical practices. Clinical decision-making should consider more than a single laboratory value. Recent efforts have developed predictive models regarding β-hCG levels, gestational age, mass size, vascularity, endometrial thickness, inflammatory markers, and other factors to improve patient selection (13–16). For instance, a scoring system proposed by Kale et al. (10) demonstrated good predictive performance for MTX failure, supporting individualized risk stratification. In our case, despite elevated β-hCG, the patient's hemodynamic stability, absence of cardiac activity, minimal free fluid, and strong fertility desire collectively justified the attempt at conservative management.

### 3.2 MTX regimen for high-β-hCG ectopic pregnancy

When conservative treatment is pursued, several MTX regimens are available. The single-dose protocol is convenient and has fewer side effects but shows lower success at higher hCG levels. The multidose protocol offers higher efficacy but requires more injections and monitoring and carries greater toxicity. The two-dose protocol strikes a balance between efficacy and convenience, with evidence suggesting superior outcomes compared with single-dose in high-risk patients (17). In our case, we employed a modified two-dose protocol with an additional third dose based on inadequate β-hCG decline by day 7, representing an individualized adaptation rather than deviation from established principles.

### 3.3 Adverse effects and monitoring

MTX is generally well-tolerated in the medical treatment of ectopic pregnancy, and most patients are able to safely complete the course of therapy. Gastrointestinal symptoms are the most common type of adverse reaction, including nausea, vomiting, abdominal pain, and stomatitis; these symptoms typically resolve on their own without requiring specific intervention. Hepatotoxicity is a well-recognized concern, with transient liver enzyme elevations frequently observed; hematological toxicity (anemia, leukopenia) is also reported (18). Baseline and serial monitoring of liver and renal function and complete blood counts are essential, particularly when multiple doses are administered. In our patient, transient liver enzyme elevation was managed successfully with hepatoprotective agents and multidisciplinary collaboration.

### 3.4 Informed consent and patient compliance

Conservative management for high-β-hCG ectopic pregnancy requires prolonged follow-up (8, 19), typically several weeks, and success depends critically on patient adherence. Real-world studies have shown that follow-up compliance can be poor (20). Therefore, thorough pre-treatment counseling and careful patient selection are paramount. Our patient's excellent adherence to the monitoring schedule and treatment regimen was fundamental to the successful outcome.

### 3.5 Role of integrated traditional Chinese medicine

The patient received multiple Traditional Chinese Medicine interventions concurrently with MTX. Although some studies suggest potential benefits of integrated Chinese and Western medicine (21), such as enhanced MTX efficacy, accelerated mass absorption, and reduced side effects, we must explicitly acknowledge that, in this single uncontrolled case, the individual contribution of TCM cannot be determined. The observed success may be attributable to MTX alone, the combined interventions, or natural history. Therefore, we view TCM as part of a multimodal approach whose components cannot be disentangled from this observation, and no causal claims can be made.

However, this plan has obvious limitations: (1) It is only applicable to selective cases without rupture and bleeding, stable mass, and stable vital signs. If abdominal pain intensifies, pelvic effusion increases, or β-HCG continues to rise sharply, surgery is required immediately; (2) Multiple MTX administrations increase the risk of adverse reactions such as liver and kidney function damage and gastrointestinal reactions, which requires enhanced monitoring and symptomatic treatment; (3) This case is a case report with a small sample size, and the safety and effectiveness of the treatment plan still need to be verified by large-sample prospective studies; (4) The course of conservative treatment is long, requiring long-term follow-up of patients, with high time cost, which is only applicable to patients with good compliance; (5) The patient's follow-up time is limited, and long-term follow-up of her subsequent fertility status has not been conducted, so it is impossible to fully evaluate the impact of integrated traditional Chinese and Western medicine conservative treatment on long-term pregnancy outcomes.

## 4 Conclusions

This case reports a patient with recurrent ectopic pregnancy after prior contralateral salpingectomy, presenting with β-hCG exceeding 5,000 IU/L, who was successfully managed conservatively with repeated MTX doses combined with integrated TCM adjunctive therapies under intensive surveillance, achieving β-hCG normalization and tubal preservation without surgical intervention. This case illustrates that in carefully selected patients, conservative management with repeated MTX may be a feasible alternative to surgery even when β-hCG exceeds conventional thresholds. Nevertheless, as a single case observation, this report cannot establish causality, and the independent contribution of Traditional Chinese Medicine to the successful outcome remains uncertain given the concurrent administration of multiple interventions. The safety and efficacy of this multimodal approach require further validation through well-designed prospective studies with larger sample sizes.

## Data Availability

The original contributions presented in the study are included in the article/supplementary material, further inquiries can be directed to the corresponding author/s.

## References

[1] Papageorgiou D Sapantzoglou I Prokopakis I Zachariou E. Tubal ectopic pregnancy: from diagnosis to treatment. Biomedicines. (2025) 13:1465. doi: 10.3390/biomedicines13061465 40564186 PMC12191334

[2] Admin A. Systemic Methotrexate in the Management of Ectopic Pregnancy and Pregnancy of Unknown Location. Annals Singapore (2023). Available online at: https://annals.edu.sg/systemic-methotrexate-in-the-management-of-ectopic-pregnancy-and-pregnancy-of-unknown-location/ (Accessed July 8, 2026). 10.47102/annals-acadmedsg.202336 38904504

[3] Lin Q Lin N Wang G Zheng X Hua R. A novel predict factor that increases the success rate of methotrexate treatment in fallopian tube pregnancy. Ann Transl Med. (2021) 9:146. doi: 10.21037/atm-20-7914 33569448 PMC7867891

[4] Kumari A Biswas R Yadav N Debnath E. Initial and day four β-hCG levels as predictors of outcome of single-dose methotrexate therapy in medical management of tubal ectopic gestation. Indian J Med Biochem. (2021). 25:91–5. doi: 10.5005/jp-journals-10054-0195

[5] American College of Obstetricians and Gynecologists' Committee on Practice Bulletins—Gynecology. ACOG practice bulletin No. 193: tubal ectopic pregnancy. Obstet Gynecol. (2018) 131:e91–103. doi: 10.1097/AOG.0000000000002560 29470343

[6] Marret H Fauconnier A Dubernard G Misme H Lagarce L Lesavre M . Overview and guidelines of off-label use of methotrexate in ectopic pregnancy: report by CNGOF. Eur J Obstet Gynecol Reprod Biol. (2016) 205:105–9. doi: 10.1016/j.ejogrb.2016.07.489 27572300

[7] NICE. Ectopic Pregnancy and Miscarriage: Diagnosis and Initial Management. London: National Institute for Health and Care Excellence (NICE) (2023). Available online at: http://www.ncbi.nlm.nih.gov/books/NBK544906/ (Accessed March 27, 2026) 31393678

[8] Elson CJ Salim R Potdar N Chetty M Ross JA Kirk Kirk EJ on behalf of the Royal College of Obstetricians and Gynaecologists. Diagnosis and management of ectopic pregnancy: green-top guideline no. 21. BJOG. (2016) 123:e15–55. doi: 10.1111/1471-0528.14189 27813249

[9] Wang X Huang L Yu Y Xu S Lai Y Zeng W. Risk factors and clinical characteristics of recurrent ectopic pregnancy: a case–control study. J Obstet Gynaecol. (2020). 46:1098–103. doi: 10.1111/jog.14253 32281241 PMC7384140

[10] Kale I Yalçinkaya C Konak G Zengin A. A new scoring system to predict the success of single-dose methotrexate therapy in ectopic pregnancy. Rev Assoc Med Bras (1992). (2025) 71:e20250639. doi: 10.1590/1806-9282.20250639 41124556 PMC12534065

[11] Abdulkareem TA Eidan SM. “Ectopic pregnancy: diagnosis, prevention and management”. In: Obstetrics. IntechOpen (2017). Available online at: https://www.intechopen.com/chapters/57672 (Accessed July 10, 2026).

[12] Slattery ME Nava HJ Spoljaric HM Gipson TD Trester R. Methotrexate use in the management of ectopic pregnancies: a retrospective study. Cureus. (2025) 17:e95826. doi: 10.7759/cureus.95826 41328090 PMC12665288

[13] Scarpelli E Capozzi VA Roberto L Gallinelli A Pezzani A Monica M . Predictors of methotrexate success and fertility outcomes in tubal ectopic pregnancy: a retrospective cohort study. Medicina (Kaunas). (2025) 61:1058. doi: 10.3390/medicina61061058 40572746 PMC12194845

[14] Cohen A Bar-On Bar-On S Cohen Y Sandel O Fouks Y Michaan N . Ruptured ectopic pregnancies following methotrexate treatment: clinical course and predictors for improving patient counseling. Reprod Sci. (2022) 29:1209–14. doi: 10.1007/s43032-022-00881-7 35157263

[15] Zorlu U Düz SA Kurtaran G Halilzade MI Elmas B. Inflammatory indices, machine learning and artificial intelligence in tubal ectopic pregnancy management. Turk J Obstet Gynecol. (2026) 23:47–55. doi: 10.4274/tjod.galenos.2026.37165 41717887 PMC12963803

[16] Levine Y Yahav L Schwarzman P Yohai D Hershkovitz R Weintraub AY. The correlation between endometrial thickness and the criteria for MTX treatment for ectopic pregnancy. J Obstet Gynaecol. (2021) 41:1230–3. doi: 10.1080/01443615.2020.1849068 33616483

[17] Pourali L Vatanchi A Mehrad-Majd H Ghomian N Jabarzadeh S. Double-dose versus single-dose methotrexate therapy in treatment of ectopic pregnancy. Curr Womens Health Rev. (2025) 21:55–61. doi: 10.2174/0115734048286695240627060120

[18] Soysal S Ilhan GA Vural M Yildizhan B Soysal S Ilhan GA . Severe methotrexate toxicity after treatment for ectopic pregnancy: a case report. Turk J Obstet Gynecol. (2016) 13:221–3. doi: 10.4274/tjod.80457 28913127 PMC5558298

[19] Sivalingam VN Duncan WC Kirk E Shephard LA Horne AW. Diagnosis and management of ectopic pregnancy. J Fam Plann Reprod Health Care. (2011) 37:231–40. doi: 10.1136/jfprhc-2011-0073 21727242 PMC3213855

[20] Bachman EA Barnhart K. Medical management of ectopic pregnancy: a comparison of regimens. Clin Obstet Gynecol. (2012) 55:440–7. doi: 10.1097/GRF.0b013e3182510a73 22510626 PMC3329644

[21] Qu HB Dengfeng W Wu T Marjoribanks J Ying S Haijun J . Chinese herbal medicine in the treatment of ectopic pregnancy. Cochrane Database Syst Rev. (2011) 2011:CD006224. doi: 10.1002/14651858.CD006224.pub3 21735404 PMC12414627

